# Integrative analysis of eQTL and GWAS summary statistics reveals transcriptomic alteration in Alzheimer brains

**DOI:** 10.1186/s12920-022-01245-5

**Published:** 2022-04-23

**Authors:** Pradeep Varathan, Priyanka Gorijala, Tanner Jacobson, Danai Chasioti, Kwangsik Nho, Shannon L. Risacher, Andrew J. Saykin, Jingwen Yan

**Affiliations:** 1 Department of BioHealth Informatics, Indiana University Purdue University Indianapolis, Indianapolis, IN USA; 2 Department of Radiology and Imaging Sciences, School of Medicine, Indiana University School of Medicine, Indianapolis, IN USA

**Keywords:** GWAS, Alzheimer’s Diseases, eQTL, HEIDI, SMR

## Abstract

**Background:**

Large-scale genome-wide association studies have successfully identified many genetic variants significantly associated with Alzheimer’s disease (AD), such as rs429358, rs11038106, rs723804, rs13591776, and more. The next key step is to understand the function of these SNPs and the downstream biology through which they exert the effect on the development of AD. However, this remains a challenging task due to the tissue-specific nature of transcriptomic and proteomic data and the limited availability of brain tissue.In this paper, instead of using coupled transcriptomic data, we performed an integrative analysis of existing GWAS findings and expression quantitative trait loci (eQTL) results from AD-related brain regions to estimate the transcriptomic alterations in AD brain.

**Results:**

We used summary-based mendelian randomization method along with heterogeneity in dependent instruments method and were able to identify 32 genes with potential altered levels in temporal cortex region. Among these, 10 of them were further validated using real gene expression data collected from temporal cortex region, and 19 SNPs from *NECTIN* and *TOMM40* genes were found associated with multiple temporal cortex imaging phenotype.

**Conclusion:**

Significant pathways from enriched gene networks included neutrophil degranulation, Cell surface interactions at the vascular wall, and Regulation of TP53 activity which are still relatively under explored in Alzheimer’s Disease while also encouraging a necessity to bind further trans-eQTL effects into this integrative analysis.

## Background

Alzheimer’s disease (AD) is the leading cause of brain dementia, along with which substantial failure of organs and mental issues arise. Accumulation of beta-amyloid plaques and tau tangles are two hallmarks of AD. The genetic mutations in genes such as ataxin-1 cause the misfolding of the proteins thereby starting a chain reaction of multiple neurodegenerative pathologies [1]. In the last decade, several large-scale genome-wide association studies (GWASs) have helped reveal mutations significantly associated with AD and the related traits. Yet, the functional mechanism through which these SNPs contribute to the development of AD remains largely unknown. This knowledge gap could be partly narrowed by investigating the effect of these SNPs on the downstream transcriptomic and proteomic levels. But the limited availability of gene and protein expression data in the brain tissue makes this a very challenging task.

Expression quantitative loci (eQTL) analysis aims to identify genetic variants that are significantly associated with the expression of one or more genes [2]. Recent findings show that most GWAS findings overlap with expression quantitative trait loci (eQTL), indicating the potential role of disease-related variants in gene regulation [3]. Although GWAS does not necessarily reveal the causal variants associated with the disease, with eQTL that links the genomic data to the transcriptomic data, one can isolate the location that potentially affect the downstream expression profile.

**Fig. 1:**
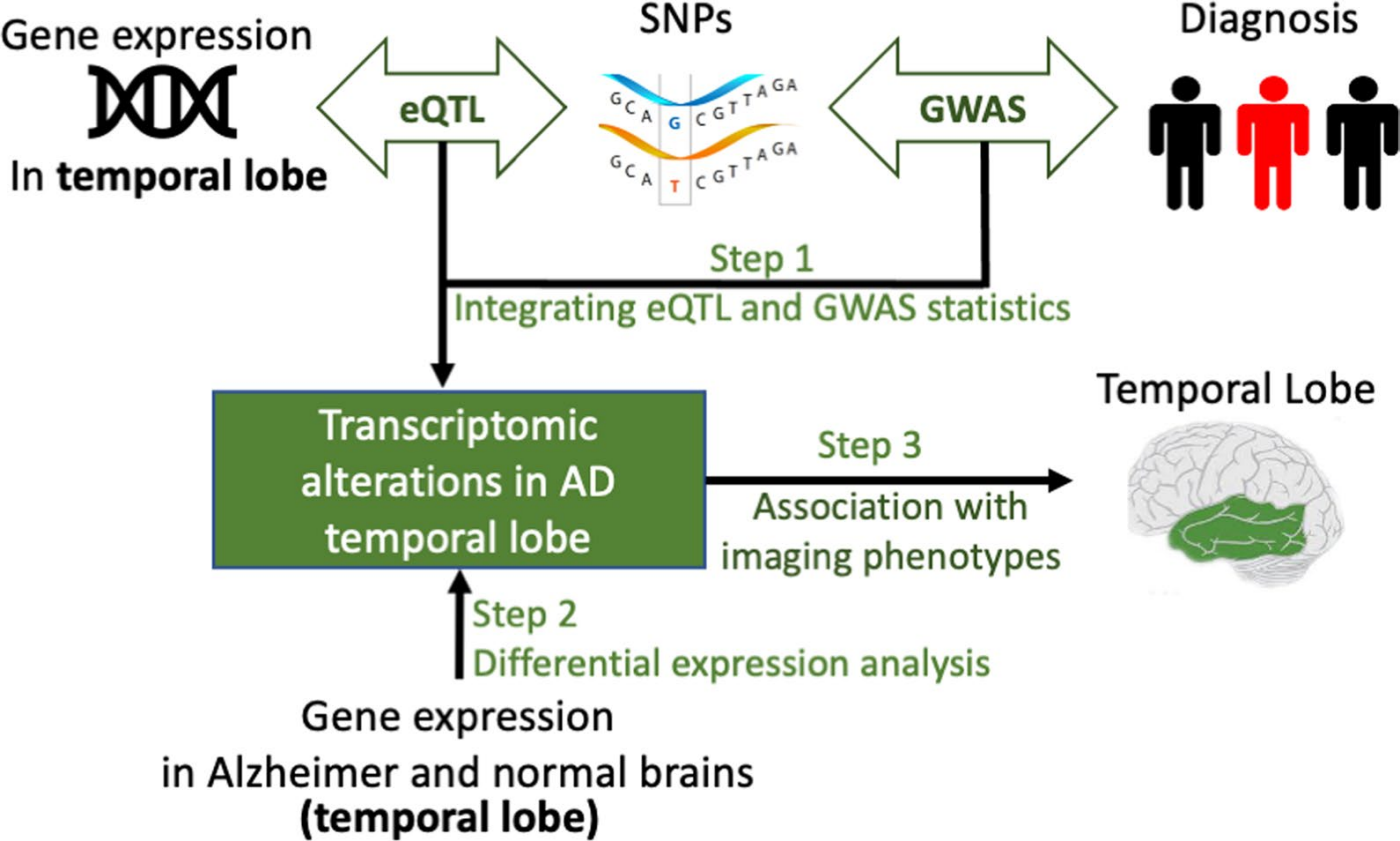
Overall pipeline of the integrative analysis of GWAS and eQTL summary statistics and the downstream validation

In this paper, leveraging the GWAS findings from International Genomics of Alzheimer’s Project (IGAP) and eQTL results of 3 brain regions from Brain eQTL Almanac (BRAINEAC), we applied a summary-based mendelian randomization method (SMR) to predict the associations between gene expression and AD in 3 brain regions of interest [4], including hippocampus, frontal cortex and temporal cortex as described in Fig. 1. While no significant gene-AD relationships were found from the hippocampus and frontal cortex, temporal cortex resulted in 37 SNPs from 32 unique genes that are significantly associated with AD in the transcriptomic level. Among these, 10 of them were found differentially expressed in AD brains when examined using the real gene expression data from temporal cortex tissue. For 37 significant SNPs associated with these 32 genes as in SMR, 19 of them were found to be associated with imaging phenotypes in temporal cortex, including FDG intensity, medial temporal lobe thickness, and lateral temporal lobe thickness. These results cement the theory that AD pathology variants have a higher influence on the temporal region of the brain. This is the first study that uses real brain expression data to validate the results obtained from the integrative analysis of GWAS and eQTL summary statistics in AD.

## Results

### Significant gene-AD associations

**Fig. 2:**
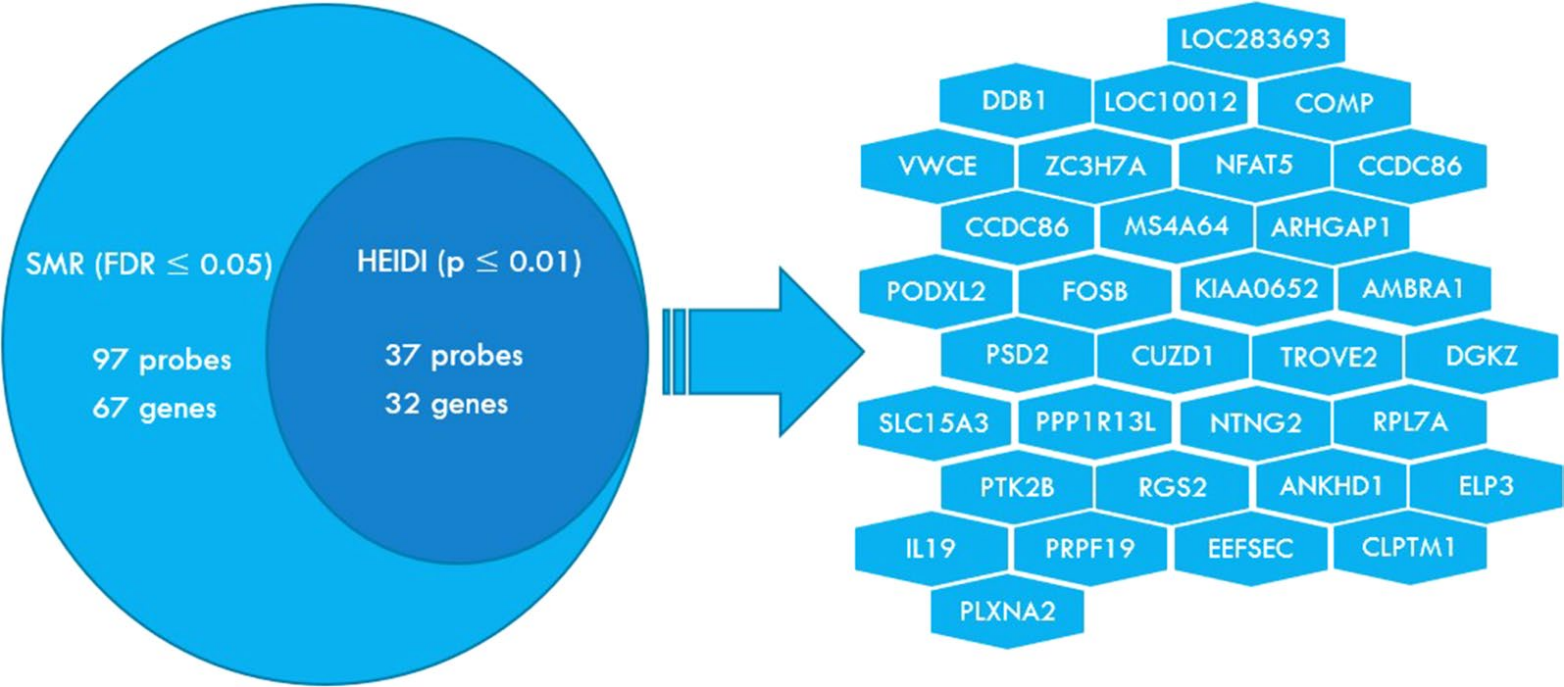
List of genes that passed both SMR and HEIDI tests in temporal cortex samples

With the GWAS summary data from the IGAP and eQTL summary data from BRAINEAC, we performed both SMR and HEIDI tests to estimate the gene-AD associations in three human brain regions: frontal cortex, temporal cortex, and hippocampal regions. For the frontal cortex and hippocampal regions, we obtained 318,168 and 195,996 probes respectively that passed the genome-wide significance threshold in the SMR test. However, after FDR correction, none of them remained significant. While both frontal cortex and hippocampal regions are known to be related to AD, our results did not show any significant transcriptomic alterations in these regions, likely due to the small sample size in eQTL study. Also, some signals might be missing since BRAINEAC now only provides cis-eQTL data. In the temporal cortex, SMR test yielded 97 significant probes with FDR corrected $p\le 0.05$ and HEIDI test returned 2224 significant probes after correction. 37 probes corresponding to 32 unique genes were found significant in both SMR and HEIDI tests as seen in Fig. 2.

### Differential gene expression in AD brains

For all 32 significant genes identified in the temporal cortex, we further compared their expression patterns between cognitively normal controls and AD using RNA-Seq data collected from the temporal cortex region in the Mayo clinic cohort. Out of 32 genes, we identified 10 of them with significant differential expression patterns in the temporal cortex region after FDR correction. Shown in Table 1 is the summary of those differentially expressed genes (DEGs).

**Table 1:** List of genes with significant differential expression levels between AD and normal brains in the temporal cortex region

Gene	logFC^a^	Corrected *p* value	logCPM^b^	LR^c^
CCDC86	− 0.2E−04	7.2E−05	11.701	15.750
CUZD1	− 0.3E−04	0.9E−05	9.634	10.850
FOSB	0.7E−05	4.1E−05	11.738	16.827
PODXL2	0.3E−05	1.7E−05	15.238	18.474
PPP1R13L	− 0.6E−04	6.9E−06	11.488	20.211
PRPF19	0.4E−05	3.1E−09	15.880	35.106
PSD2	− 0.7E−04	6.9E−05	16.418	33.557
PTK2B	0.4E−05	3.1E−05	16.594	17.366
RGS2	0.2E−05	0.1E−05	13.428	6.514
SLC15A3	− 0.7E−04	3.9E−09	12.117	34.668

^a^The log of fold-change that describes the difference of expression between groups

^b^The log of counts-per-million that describes the expression level of each gene

^c^Likelihood ratios of the genes

### Pathways and networks enriched in temporal cortex

**Fig. 3:**
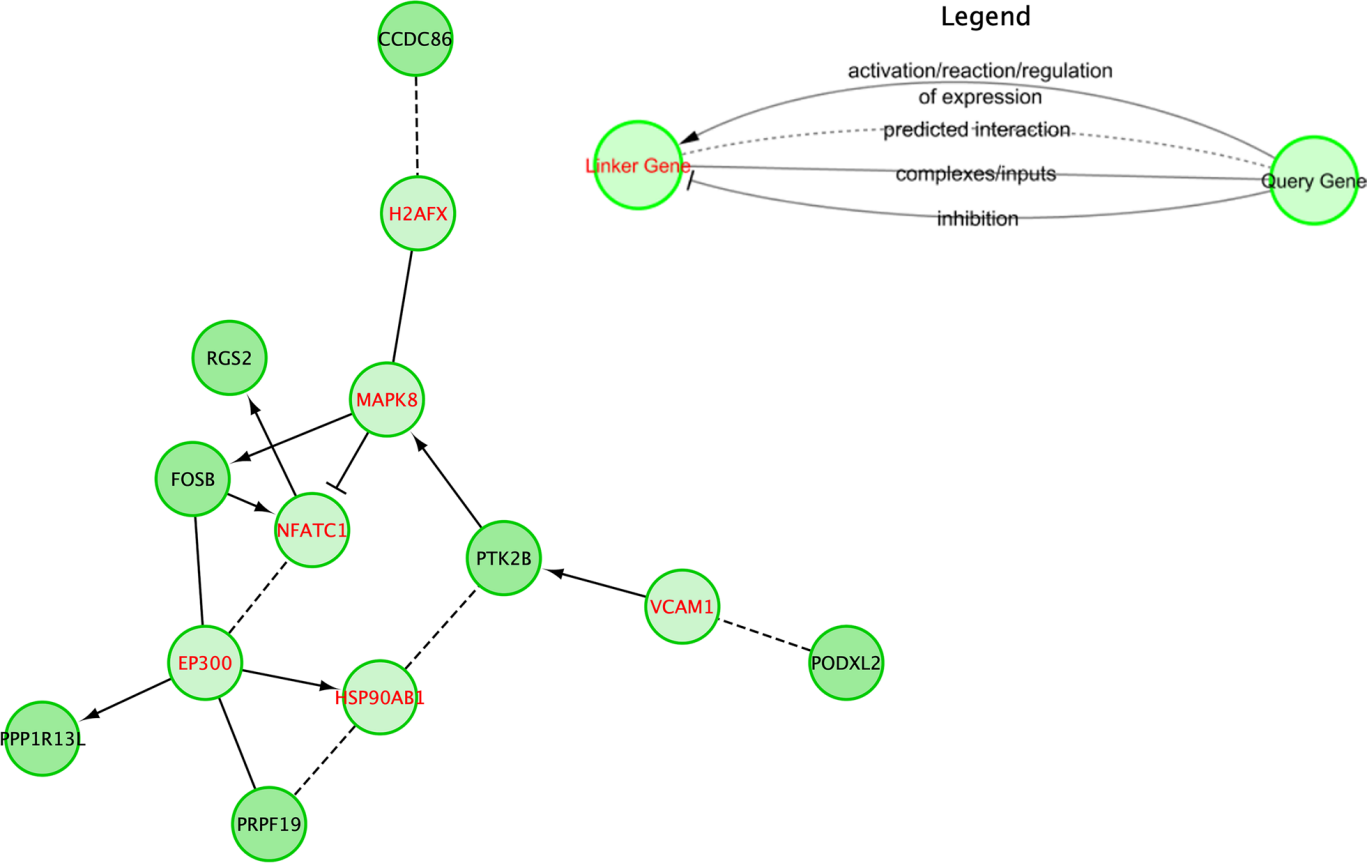
Top pathways enriched by 10 significant genes

**Fig. 4:**
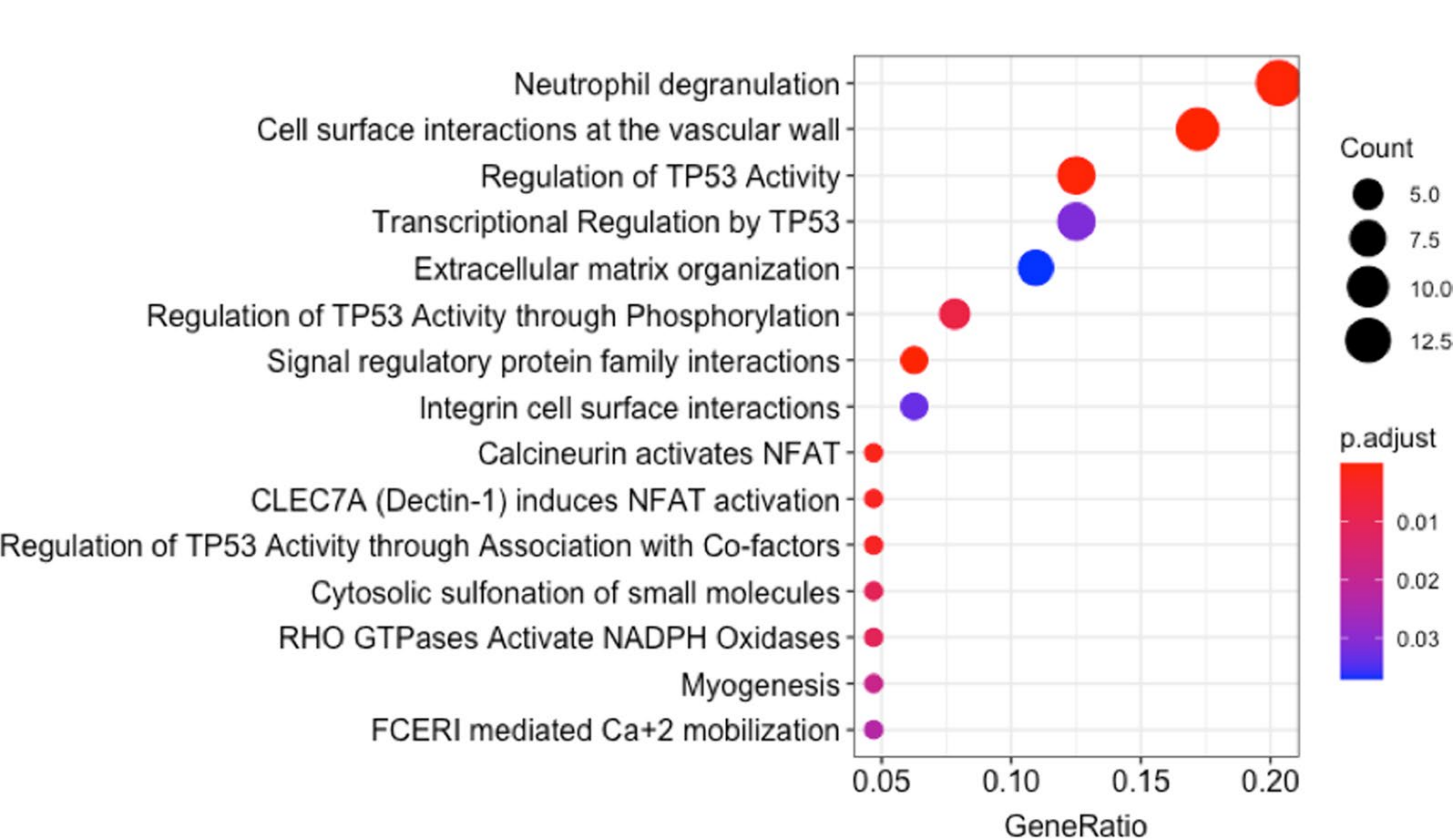
Functional interactions among 10 significant genes that passed SMR, HEIDI tests, and further showed differential expression patterns in independent cohort

Using RectomeFA, 15 pathways in REACTOME database were found to be enriched by those 10 significant genes. Shown in Fig. 3 is a list of those pathways and the ratio between the number of significant genes and all member genes in the pathway. We further examined the interactions between those 10 genes by mapping them to the REACTOME protein interaction network. We used the ReactomeFI in Cytoscape to investigate the direct or indirect interactions between these genes. Out of 10 genes, 7 of them were found to be connected with 6 intermediate or linker genes (Fig. 4). In the subnetwork, *EP300* and *MAPK8* were observed as two hub genes with the highest connectivity, and *PTK2B* has the highest connectivity among those 10 significant genes.

### Association with temporal cortex phenotypes

**Fig. 5:**
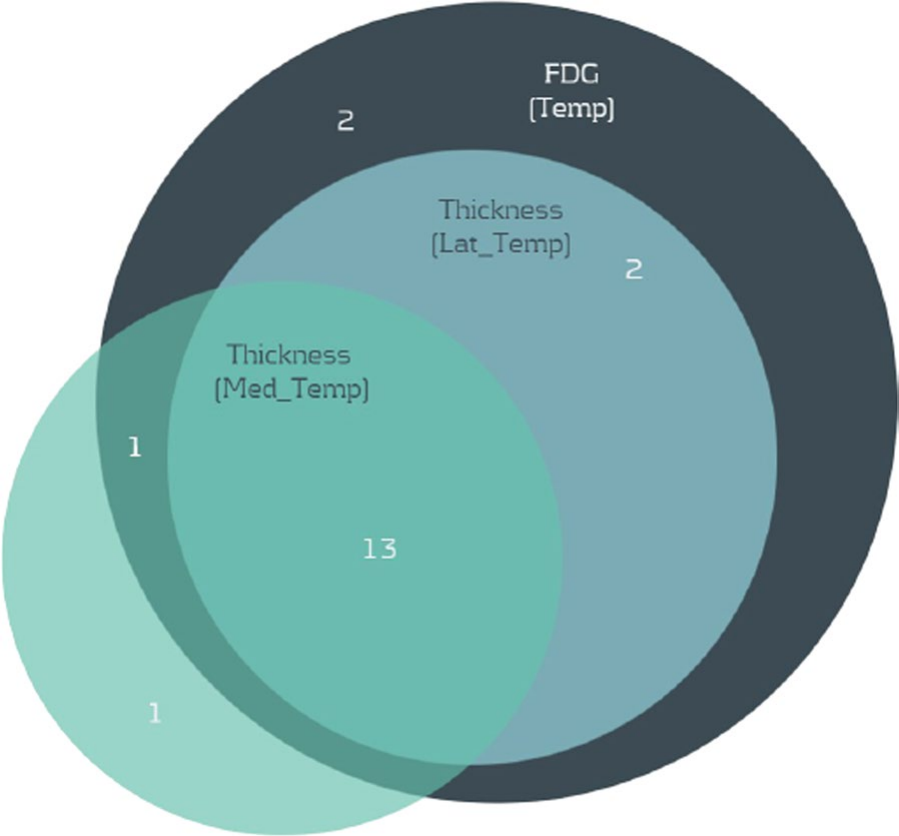
Number of significant SNPs associated with three imaging phenotypes of temporal lobe

When examined the original GWAS and eQTL summary statistics, the significant association of those 10 genes with AD disease status was found to come from 11 unique SNPs. Using these 11 SNPs as seeds, we expanded our SNP set by including other neighboring SNPs located within the same LD block. In total, 613 SNPs were found and 518 of them were identified with genotype data from the ADNI cohort. Then, we tested their association with three AD-related brain imaging phenotypes of the temporal cortex region in PLINK. Linear regression models were used to examine the association between each pair of SNP and brain imaging phenotype. After FDR correction, our association analysis identified 18 SNPs significantly associated with FDG intensity levels, 15 SNPs associated with the thickness of the medial temporal lobe, and 15 SNPs for the lateral temporal lobe (Table 2). In total, there are 19 significant SNPs, all from chromosome 19, and 13 of them were found to be associated with all three imaging phenotypes (Fig. 5). These SNPs are all located with two LD blocks of rs73050293 and rs7669277 in *NECTIN* and *TOMM40* respectively. 11 of the SNPs were intron variants while two other SNPs, rs11556505 and rs15758 identified as synonymous variants located in the coding sequence. Most of the SNPs in *NECTIN* are genic downstream transcript variants and intron variants, while one SNP rs71352238 belongs to the category of upstream transcript variant, which might be a part of the gene regulation.The intronic variants can be responsible for regulating the gene expression since there has been multiple reports of miRNAs, siRNAs, piwi-interacting RNAs (piRNAs), long noncoding RNAs (lncRNAs), and small nucleolar RNAs (snoRNAs), which do have regulatory effect in transcription , to be present in the intronic region. Variation in this region could lead to differential regulation of the gene transcription [5]. For a more detailed analysis of the intronic variants, we used the SNP Nexus online tool [6–10]. We observed that under Ensemble Regulatory Build [11], about 88 percentage of the variants feature type belonged to promoter region and was active in brain and brain related epigenomes. While the other variant feature types belong to CTCF binding sites or open chromatin sites, they were also labelled to be inactive for the above mentioned epigenomes. As for the ENCODE database results [12], it was seen that H3K36me3, H3K4me1 and, H3K4me3 histone feature and DNase open chromatin feature were the most prominent features where these variants were present. This could also imply that these variants might play a role in Alzheimer’s disease by transcriptomic regulation of the epigenetic factors.

**Table 2:** Significant associations between SNPs and 3 distinct imaging phenotypes of temporal cortex region

SNP	FDG phenotype	Lateral temporal lobe thickness	Medial temporal lobe thickness
rs34278513	0.004	0.035	0.445
rs412776	0.006	0.036	0.445
r73050293	0.069	0.164	0.028
rs3865427	0.006	0.068	0.445
rs12972156	1.7E−08	0.005	0.007
rs12972970	1.7E−08	0.003	0.007
rs3432646	1.7E−08	0.003	0.007
rs283815	2.2E−08	0.009	0.001
rs6857	3.2E−10	0.002	0.0003
rs71352238	1.7E−08	0.002	0.007
rs184017	1.8E−08	0.009	0.001
rs15781	0.0002	0.078	0.211
rs2075650	1.7E−08	0.002	0.007
rs15781	2.2E−08	0.009	0.001
rs34095326	0.0003	0.117	0.039
rs34404554	1.7E−08	0.002	0.007
rs11556505	1.7E−08	0.002	0.007
rs157582	2.2E−08	0.009	0.001
rs59007384	1.7E−08	0.009	0.001

The top three significant pathways enriched by 10 significant genes are Neutrophil degranulation, Cell surface interactions at the vascular wall, and Regulation of *TP53* activity. Further probing of these pathways, neutrophils were found to migrate around AB proteins and in absence of neutrophils, improved cognitive functions, impeded microgliosis and $\alpha \beta$1–42 levels in brain homogenates [13]. Neutrophil downregulation has provided a reducing effect on levels of phosphorylated tau proteins. The secretory and the azurophil neutrophil granules have a common component, *CAP37* protein, which is also seen upregulated in AD. This protein has been positively correlated with the AB-RAGE signaling pathway and also proven to activate monocytes by varying the cell adhesion pathway expression profile. Co-incidentally, we have cell surface interactions with vascular walls as one of the top significant pathways, where the process of extravasation is carried out by the vascular endothelial cells attached with neutrophils. The other important process that tangles these two pathways is NETosis (Neutrophils extracellular traps), wherein the neutrophils bind with the blood vessels and become mobile enough to target the parenchyma cells, leading to their cell death [14]. Studies have shown that NET depletion has provided positive feedback on reduced memory loss and other neuropathological features. Few studies have pointed out neutrophil levels as a potential indicator of cognitive decline [15]. Further, the proteins that belong to the Neutrophil pathway are significantly associated with Chr19, which also coincides with the location of all the significant SNPs associated with the imaging phenotype.

Regulation of TP53 is the other significant pathway along with the above two. Studies have shown that approximately 2-fold upregulation of p53 has been recorded in the superior temporal gyrus of Alzheimer’s patients. It has also been found to phosphorylate tau proteins in HEK cells [16]. Furthermore, p53 has been seen to aggregate and interact with tau oligomers in AD patients [17]. Thus, our exploration adds weight to the cause of relating AD and the cancerous pathways involving p53.

## Conclusion

We performed an integrative analysis of AD GWAS and brain eQTL summary statistics to estimate the potential transcriptomic changes inside AD brains. Using real RNA-Seq gene expression data inside corresponding brain regions, identified genes with potential association with AD were further examined for altered expression patterns in AD brains. Significant gene-AD associations were found only in the temporal cortex region, but not in the frontal cortex and hippocampal regions. SNPs associated with two significant genes *TOMM40* and *NECTIN*, as in the original eQTL summary, also showed significant association with the FDG intensity level and thickness of the temporal lobe. Further pathway and network analysis provided an enriched pathway profile, with neutrophil degranulation, Cell surface interactions at the vascular wall, and Regulation of TP53 activity as the most significant ones. Further efforts are warranted to investigate the association of neutrophils with AD. With the validation from real expression data collected from brain regions, the results of this study confirmed the potential of integrative GWAS and eQTL analysis in exploring the transcriptomic changes when lack of tissue-specific expression data. Further efforts to explore new merging methods for integrative analysis and trans-eQTL effects are warranted in the future work.

## Data and methods

### GWAS summary statistics

GWAS summary statistics were downloaded from the International Genomics of Alzheimer’s Project (IGAP). IGAP is a large two-stage study based upon genome-wide association studies (GWAS) on individuals of European ancestry. In stage 1, IGAP used genotyped and imputed data on 7,055,881 single nucleotide polymorphisms (SNPs) to meta-analyze four previously-published GWAS datasets consisting of 17,008 Alzheimer’s disease cases and 37,154 controls (The European Alzheimer’s disease Initiative—EADI the Alzheimer Disease Genetics Consortium—ADGC The Cohorts for Heart and Aging Research in Genomic Epidemiology consortium—CHARGE The Genetic and Environmental Risk in AD consortium—GERAD). In stage 2, 11,632 SNPs with $p \le 10^{-6}$ were genotyped and tested for association in an independent set of 8572 Alzheimer’s disease cases and 11,312 controls. Finally, a meta-analysis was performed combining results from stages 1 and 2 [18].

### EQTL summary data

EQTL summary statistics from the Brain eQTL Almanac (BRAINEAC) were downloaded through the UK Brain Expression Consortium (UKBEC). In total, there were 134 postmortem brains included in the study. RNA from ten brain regions were extracted and analyzed with Affymetrix Human Exon 1.0 ST and eQTLs were classified and grouped by marker type, expression type, and cis/trans type. Our study focused on three brain regions including the temporal cortex, frontal cortex, and hippocampal regions, in which the gene expression data are available in the AD brains through the AMP-AD knowledge portal.

### SMR and HEIDI test

SMR (Summary-based Mendelian Randomization) software was used to integrate summary-level data from the IGAP GWAS with data from BRAINEAC eQTL studies to identify genes with potential expression levels altered in certain brain regions of AD brains [19]. Individual-level SNP genotype data from 1000 Genomes European population were used to estimate linkage disequilibrium (LD) block information. Following the default settings, SMR analysis was performed on cis-regions with a window of 2000 Kb, and LD r-squared threshold range was set between 0.9 ± 0.05. Note that the association observed in the SMR test does not necessarily indicate that gene expression and AD are affected by the same underlying causal variant. The association could be due to the top associated eQTL being in LD with two causal variants, one affecting gene expression and the other affecting AD. Compared to such linkage effect, pleiotropy effect is of more interest where gene expression and a trait (e.g., AD) share the same causal variant. Therefore, we further applied HEIDI (heterogeneity in dependent instruments) test to differentiate pleiotropy from linkage. Following the [4], we used a genome-wide significance level ($p\le 8.4 \times 10^{-6}$) for SMR test and *p* value threshold of 0.01 for the HEIDI test. Subsequent analyses are focused on gene-AD associations that passed both SMR and HEIDI tests.

### Differential expression analysis

For all the genes that passed both SMR and HEIDI tests, we further compared their expression levels between cognitively normal controls and AD patients using RNA-Seq data from the corresponding brain tissues. In our case, significant gene-AD associations were only detected in the temporal cortex region. So we downloaded the RNA-Seq data from the temporal cortex tissue in the Mayo Clinic cohort [20]. The differential expression analysis was performed using the R package, EdgeR [21, 22], where baseline age, sex, batch, RIN, and *APOE* e4 status were used as covariates. EdgeR is one of the most robust packages for differential expression analysis, which uses a generalized linear model (GLM) approach based upon negative binomial distribution. Normalization of the RNA-Seq data was performed using Trimmed mean of M values (TMM method). Before analysis, genes were filtered with counts per million as very low counts provide little evidence for differential expression. *p* Values obtained from differential expression analysis were adjusted using the FDR method and a hard threshold was set at 0.05.

### Pathway and network enrichment analysis

We further performed pathway enrichment analysis for all genes that passed the SMR, HEIDI, and differential expression tests. The ReactomePA package in R was used to perform the enrichment analysis based on the pathways in the Reactome database [23]. This package provides the best pathways for each gene-set from the input list of genes as well as the membership of genes to each pathway. Besides, we also mapped these 10 genes to the protein interaction network using ReactomeFI in Cytoscape to examine their direct or indirect functional interactions in the pathway. [24]

### Genetic association analysis with brain imaging Phenotype

With the significant gene-AD associations identified from the temporal cortex, we further examined the association of these genes with the brain imaging phenotypes in the temporal cortex region. We downloaded the FDG intensity levels and mean thickness of the temporal cortex regions from the ADNI cohort. The initial phase (ADNI-1) was launched in 2003 to test whether serial magnetic resonance imaging (MRI), positron emission tomography (PET), other biological markers, and clinical and neuropsychological assessment could be combined to measure the progression of MCI and early AD. ADNI-1 was extended to subsequent phases (ADNI-GO, ADNI-2, and ADNI-3) for follow-up for existing participants and additional new enrollments. More information about the data collection and preprocessing steps can be found at www.adni-info.org [25, 26]. In total, we studied 3 imaging phenotypes, including the overall FDG intensity level of the temporal lobe region and the mean thickness of medial and lateral temporal regions. To remove the potential bias introduced by confounding factors, baseline age, gender, and education years were included as covariates to adjust FDG intensity levels. Intracranial volume (ICV) was used as an additional covariate for thickness measures. The association between eQTL SNPs of those significant genes and 3 imaging phenotypes was tested using PLINK. LD information derived from 1000 Genomes using European population were used for FDR correction of the SNP *p* values in each phenotype.

## Data Availability

The data that support the findings of this study are available from the AMP-AD knowledge portal and the ADNI but restrictions apply to the availability of these data. Data are however available from the AMP-AD knowledge portal and the ADNI upon reasonable application.

## Abbreviations

AD: Alzheimer’s disease
SNP: Single nucleotide polymorphism
GWAS: Genome wide association studies
eQTL: Expression quantitative trait loci
IGAP: International genomics of Alzheimer’s project
BRAINEAC: Brain eQTL Almanac
SMR: Summary-based Mendelian randomization
DEG: Differentialy expressed genes
miRNA: MicroRNA
siRNA: Small interfering RNA
piRNA: Piwi-interacting RNA
lncRNA: Long non-coding RNA
snoRNA: Small nucleolar RNA
AB: Alpha-beta
NET: Neutrophils extracellular trap
HEK: Human embryonic kidney
UKBEC: UK brain expression consortium
AMP-AD: Accelerating medicines partnership program for Alzheimer’s disease
GLM: Generlaized linear model
TMM: Trimmed mean of M values
FDR: False discovery rate
FDG: Fludeoxyglucose
MRI: Magnetic resonance imaging
PET: Positron emission tomography
ICV: Intracranial volume
